# Increased antipsychotic drug concentration in hospitalized patients with mental disorders following COVID-19 infection: a call for attention

**DOI:** 10.3389/fpsyt.2024.1421370

**Published:** 2024-07-15

**Authors:** Rui Yang, Jin-Ling Wan, Chen-Qi Pi, Tian-Hui Wang, Xue-Quan Zhu, Shuang-Jiang Zhou

**Affiliations:** ^1^ Beijing Anding Hospital, Capital Medical University, Beijing, China; ^2^ Zhangjiakou Shalingzi Hospital, Zhangjiakou Mental Health Center, Zhangjiakou, Hebei, China; ^3^ Psychiatry Department, Shunyi Women’s & Children’s Hospital of Beijing Children’s Hospital, Beijing, China; ^4^ Beijing HuiLongGuan Hospital, Peking University HuiLongGuan Clinical Medical School, Beijing, China

**Keywords:** coronavirus disease-2019, antipsychotic concentration, mental disorder, therapeutic drug monitoring, clozapine

## Abstract

**Purpose:**

Examine the alterations in antipsychotic concentrations following coronavirus disease-2019 (COVID-19) infection among hospitalized patients with mental disorders and conduct an analysis of the factors influencing these changes.

**Methods:**

Data were collected from inpatients at Beijing Huilongguan Hospital between December 12, 2022, and January 11, 2023, pre- and post-COVID-19. Based on the Diagnostic and Statistical Manual of Mental Disorders, Fifth Edition, 329 inpatients with mental disorders were included (3 with incomplete data excluded). Primary outcomes assessed changes in antipsychotic concentrations pre- and post-COVID-19, while secondary outcomes examined factors linked to concentration increases and antipsychotic dose adjustments.

**Results:**

Clozapine (P < 0.001), aripiprazole (P < 0.001), quetiapine (P = 0.005), olanzapine (P < 0.001), risperidone (P < 0.001), and paliperidone (P < 0.001) concentrations increased post-COVID-19 in patients with mental disorders. Notably, clozapine concentration surpassing pre-infection levels was highest. Clozapine users were more likely to adjust their dose (50.4%) compared to olanzapine (17.5%) and other antipsychotics. Moreover, traditional Chinese patent medicines and antibiotics during COVID-19 infection were associated with antipsychotic reduction or withdrawal (OR = 2.06, P = 0.0247; OR = 7.53, P = 0.0024, respectively).

**Conclusion:**

Antipsychotic concentrations in hospitalized patients with mental disorders increased after COVID-19 infection, that may be related not only to COVID-19, but also to the use of Chinese patent medicines during infection. The pre-infection concentration and types of antipsychotics, patient’s gender, and combination of traditional Chinese medicine or antibiotics, were factors found to correlate with increased drug concentrations and necessitate dose adjustments.

## Introduction

In 2019, with the rapid spread of the coronavirus disease-2019 (COVID-19) worldwide, the World Health Organization announced that this will be an extremely serious public health event, posing a huge threat to the health and safety of people worldwide. Patients with mental disorders constitute a vulnerable group in society and should be provided more attention and care when facing the disease. Especially for patients diagnosed with severe mental disorders pre-infection, the risk of infection, disease severity, and post-infection mortality are significantly increased (1). The COVID-19 epidemic has had a serious negative impact on the lives and treatment of people with mental disorders (2–4). Moreover, the ensuing psychological pressure has produced many adverse factors for disease development (5, 6).

Because of the disease characteristics of patients with mental disorders, they need to take antipsychotics for a long time to maintain a normal life, and the stability of their concentration is undoubtedly an important factor in maintaining disease stability (7). Few cases have been reported globally in patients with mental disorders who received significantly elevated clozapine concentrations after COVID-19 (8–10). Additionally, studies have demonstrated that antipsychotics may exacerbate COVID-19 symptoms, which are mainly related to clozapine-induced granulocytopenia, a side effect of the drug (11, 12). Therefore, concentration control should be performed with caution. Simultaneously, an expert consensus on the use of clozapine during the COVID-19 pandemic recommends that the clozapine dosage be halved in patients with COVID-19 to avoid drug toxicity at elevated concentrations and reduce the occurrence of side effects (13). A Canadian study has reported that patients with mental disorders after COVID-19 had varying degrees of adjustment to antipsychotic medication (14).

Clozapine metabolism is mainly mediated by the cytochrome CYP1A2 enzyme (15) which is inhibited by inflammation, leading to a significant increase in clozapine levels during infection (16). In addition to clozapine, various first-line antipsychotics are used, most of which are also involved in biological transformation by P450 enzymes (17, 18). Therefore, antipsychotics may interact with the infection and treatment of COVID-19, resulting in changes in the drug concentration, which may affect drug efficacy and safety. However, no studies have analyzed the correlation between the concentrations of other antipsychotics and COVID-19. Therefore, the present study mainly focused on changes in antipsychotic concentrations mediated by the P450 enzymes (including clozapine, quetiapine, olanzapine, aripiprazole, risperidone, and paliperidone). We aimed to investigate the impact of COVID-19 infection on antipsychotic concentrations in patients with mental disorders, with the goal of providing corresponding recommendations for adjusting therapy regimens and monitoring plasma levels of antipsychotics.

## Materials and methods

### Participants

In this study, the patients with mental disorders who were hospitalized at Beijing Huilongguan Hospital and infected with COVID-19 during their hospitalization from December 12, 2022 to January 11, 2023 were retrospectively selected. All participants were from the COVID-19-infected areas of the hospital. Patients in the infected areas need to be isolated and restricted in their range of activities. According to the following inclusion and exclusion criteria, a total of 329 patients were enrolled in the study.

The inclusion criteria were as follows: (1) patients diagnosed with mental disorders, including schizophrenia, bipolar disorder, and other mental disorders, by trained psychiatrists utilizing the Diagnostic and Statistical Manual of Mental Disorders, Fifth Edition diagnostic criteria, who are undergoing treatment with antipsychotic medications; (2) confirmed COVID-19 (according to the diagnostic criteria in the Diagnosis and Treatment Protocol for Novel Coronavirus Pneumonia [Trial Version 9] in China (19), the patient is confirmed to be infected with COVID-19 based on medical history, clinical symptoms, Polymerase Chain Reaction or rapid antigen test [Zhuhai Lizhu Reagent Co., Guangdong, China]); (3) COVID-19 occurring after the onset of the mental disorder; (4) patient’s psychiatric symptoms were well controlled, and doses were maintained the same between two times of therapeutic drug monitoring (TDM).

The exclusion criteria were as follows: (1) mental disorders caused by physical diseases (serious internal organ diseases, liver and renal insufficiency); (2) drug-induced mental disorders; (3) infection caused by other viruses or bacteria; (4) lactating or pregnant women; (5) involuntary patients.

### Procedures

#### General data collection

General demographic information collected included age, sex, family history, smoking status, and underlying diseases. The clinical data related to mental illness included diagnosed illness, age at first onset, total duration of illness, and medication (type of antipsychotic used, whether it was combined with other psychotropic drugs, drugs it was combined with, daily dose of the main antipsychotic, whether it was reduced, and daily dose after reduction).

#### Collection of clinical data related to COVID-19

The post-infection clinical symptoms of the patients, including 11 major and other symptoms, were collected according to the Diagnosis and Treatment Protocol for Novel Coronavirus Pneumonia (Trial Version 9) in China (19). Information on the highest body temperature within 14 days post-infection and hematological analysis results (including white blood cells and lymphocytes) within 14 days of infection was also collected.

#### Drug concentration

At baseline, the patients’ drug concentrations within 1 month prior to infection were collected (for patients prescribed antipsychotics, we routinely monitor drug concentrations on a monthly basis to assess therapeutic adherence and ensure safety; and patients who did not adjust their drug dosage were screened out based on diagnosis and treatment records). Four milliliters of venous blood was extracted and collected using a disposable vacuum sampling vessel (without additives). After centrifugation, serum was collected. And its concentration was determined by latex immunonephelometry using a Beckman Coulter AU5800 biochemical analyzer (Beckman Coulter, Inc., Brea, CA, USA).

To collect drug concentrations post-infection, measurements were taken 14 days after the onset of COVID-19 symptoms. Most patients experienced significant remission of symptoms by this time (20).

Reference standard for concentration range: According to the *Consensus Guidelines for Therapeutic Drug Monitoring in Neuropsychopharmacology: Update 2017* published by the Arbeitsgemeinschaft für Neuropsychopharmakologie und Pharmakopsychiatrie (AGNP) in 2018 (21), the reference ranges of concentration were as follows: clozapine, 350–600 ng/mL; olanzapine, 20–80 ng/mL; quetiapine, 100–500 ng/m:; aripiprazole and dehydroaripiprazole, 150–500 ng/mL; risperidone, 20–60 ng/mL (risperidone and 9-hydroxyrisperidone), and paliperidone, 20–60 ng/mL (9-hydroxyrisperidone).

### Analytic strategy

Descriptive statistical analyses were performed on the general demographic and clinical data. Age, age at first onset, daily dose of antipsychotics used pre- and post-infection, concentration pre- and post-infection, rate of change of concentration, and time from monitoring of concentration to symptom onset are represented as means and standard deviations. Statistical data on sex, body temperature, and concentration range pre- and post-infection are expressed as numbers (n) and proportions. Serum concentrations of clozapine, aripiprazole, olanzapine, quetiapine, risperidone, and paliperidone were compared pre- and post-infection, the t test was used for data conforming to normal distribution, and the rank sum test was used for data not conforming to normal distribution.

Considering that the reference ranges of concentration of different drugs are quite different, there was no obvious significance in simply comparing the figures with elevated concentrations. Meanwhile, the supratherapeutic concentrations is more meaningful for reduce or discontinue the dosage. Therefore, in this study, we used the concentration reference range as the uniform reference standard. According to AGNP, concentrations of antipsychotic were divided into three levels, subtherapeutic concentrations, within the range of therapeutic concentrations and supratherapeutic concentrations for comparison between pre- and post-infection. And the formula “(Infection_TDM – Baseline_TDM) ÷ Baseline_TDM x 100%” was used to calculate the percentage of patients whose antipsychotic concentrations post-infection exceed the concentration range pre-infection. The chi-square test was used to compare different antipsychotic concentrations. Based on the AGNP, the concentration was divided into two categories: below or equal to and above the recommended range, which was the dependent variable. A logistic regression analysis was conducted to determine whether the drug dose was adjusted as a dependent variable, individual factors such as age, sex, and drug type were the independent variables, and the association between the independent and dependent variables was analyzed.

All data were analyzed using the Statistical Package for the Social Sciences version 26.0, and significance was set at P < 0.05. Participants with incomplete data were excluded from the statistical analysis.

### Ethical consideration

The research has been approved by the Ethics Committee of Beijing HuiLongGuan Hospital (approval number: 2022–22-KE), and informed consent was signed by each participant. This study was conducted in accordance with the Declaration of Helsinki.

## Results

### Study population and characteristics

In total, 329 patients with mental disorders were included in this study. Three patients with incomplete data were excluded, and 326 patients were analyzed. The overall age of enrolled patients was 57.0 ± 14.5 years. According to the recommended AGNP concentration range, the percentage of patients whose antipsychotic concentrations post-infection exceed the concentration range pre-infection were: aripiprazole (25.00 ± 38.00%), olanzapine (15.70 ± 26.80%), risperidone (24.00 ± 32.60%) clozapine (46.00 ± 60.77%) paliperidone (31.80 ± 33.70%) quetiapine (26.00 ± 45.90%); supratherapeutic concentration values were reported in 9 (45%) aripiprazole-, 63 (52.5%) olanzapine-, 14 (56.0%) risperidone-, 25 (41.6%) clozapine-, 5 (45.5%) paliperidone-, 2 (8.8%) quetiapine-treated patients post-infection. Due to increased antipsychotic concentrations, the patient number of reduced or discontinued dosage was aripiprazole 5 (25.0%), olanzapine 21 (17.5%), risperidone 4 (16.0%), clozapine 63 (50.4%), paliperidone 3 (27.3%), quetiapine4 (16.0%). It can be observed that clozapine has the highest proportion among them (**Table 1**).

**Table 1:** Medical history, therapeutic drugs, and drug concentrations of patients using different antipsychotics.

Variables	Aripiprazole	Olanzapine	Risperidone	Clozapine	Paliperidone	Quetiapine
N	20	120	25	125	11	25
Age (y)	49.50 ± 19.67	55.23 ± 15.37	60.76 ± 9.92	59.23 ± 11.79	56.36 ± 15.16	57.32 ± 19.16
Age at onset (y)	26.85 ± 11.19	25.84 ± 10.90	26.20 ± 8.91	24.96 ± 8.54	23.45 ± 9.50	34.32 ± 19.71
Total disease course (y)	22.45 ± 16.18	29.08 ± 14.51	34.40 ± 12.73	33.94 ± 12.73	32.64 ± 15.56	23.00 ± 15.30
Dosage pre-infection (mg)	19.50 ± 6.86	15.00 ± 4.78	4.56 ± 1.33	201.00 ± 81.41	7.91 ± 3.62	345.00 ± 222.09
Dosage post-infection (mg)	18.13 ± 6.83	13.99 ± 5.06	4.12 ± 1.67	153.40 ± 82.75	7.09 ± 2.77	327.00 ± 230.37
Time between hospitalization and TDM (d)	117.40 ± 154.92	138.42 ± 194.60	98.96 ± 157.54	239.30 ± 352.24	126.45 ± 108.55	86.84 ± 70.83
Concentration pre-infection (ng/ml)	332.54 ± 184.26	58.81 ± 26.44	45.14 ± 22.14	371.25 ± 179.57	31.58 ± 17.07	123.23 ± 96.84
Concentration post-infection (ng/ml)	487.36 ± 224.33	84.07 ± 30.66	71.36 ± 36.16	596.61 ± 342.75	53.54 ± 27.20	203.99 ± 173.03
Exceed pre-infection concentration range (%)	25.00 ± 38.00	15.70 ± 26.80	24.00 ± 32.60	46.00 ± 60.77	31.80 ± 33.70	26.00 ± 45.90
Time between TDM and onset of infection (d)	6.68 ± 4.36	7.18 ± 4.57	9.52 ± 3.56	4.27 ± 3.92	9.90 ± 4.00	8.36 ± 4.66
Sex						
Male	12 (60.0%)	91 (75.8%)	22 (88.0%)	77 (61.6%)	8 (72.7%)	15 (60.0%)
Female	8 (40.0%)	29 (24.2%)	3 (12.0%)	48 (38.4%)	3 (27.3%)	10 (40.0%)
Temperature						
< 37.3 (°C)	4 (20.0%)	43 (35.8%)	7 (28.0%)	40 (32.0%)	6 (54.6%)	12 (48.0%)
≥ 37.3 (°C)	16 (80.0%)	77 (64.2%)	18 (72.0%)	85 (68.0%)	5 (45.4%)	13 (52.0%)
Baseline_TDM						
Lower	3 (15.0%)	3 (2.5%)	4 (16.0%)	66 (52.8%)	3 (27.3%)	14 (56.0%)
Normal	12 (60.0%)	88 (73.3%)	15 (60.0%)	47 (37.6%)	8 (72.7%)	11 (44.0%)
Higher	5 (25.0%)	29 (24.2%)	6 (24.0%)	12 (9.6%)	0 (0.0%)	0 (0.0%)
Infection_TDM						
Lower	0 (0.0%)	2 (1.7%)	2 (8.0%)	34 (27.2%)	2 (18.2%)	9 (36.0%)
Normal	11 (55.0%)	55 (45.8%)	9 (36.0%)	39 (31.2%)	4 (36.4%)	14 (56.0%)
Higher	9 (45.0%)	63 (52.5%)	14 (56.0%)	52 (41.6%)	5 (45.5%)	2 (8.0%)
Mental disorders						
Schizophrenia	13 (65.0%)	98 (81.7%)	25 (100.0%)	117 (93.6%)	11 (100.0%)	12 (48.0%)
Bipolar disorder	1 (5.0%)	14 (11.7%)	0 (0.0%)	6 (4.8%)	0 (0.0%)	2 (8.1%)
Others	6 (30.0%)	8 (6.7%)	0 (0.0%)	2 (1.6%)	0 (0.0%)	11 (44.0%)
Concomitant medications						
Antidepressant	2 (10.0%)	6 (5.0%)	1 (4.0%)	8 (6.4%)	0 (0.0%)	4 (16.0%)
Valproate/Lamotrigine	2 (10.0%)	23 (19.2%)	0 (0.0%)	11 (8.8%)	1 (9.1%)	6 (24.0%)
Lithium	0 (0.0%)	7 (5.8%)	0 (0.0%)	2 (1.6%)	1 (9.1%)	0 (0.0%)
Benzodiazepine	7 (35.0%)	35 (29.2%)	3 (12.0%)	23 (18.4%)	1 (9.1%)	9 (36.0%)
Reduction/discontinuation ratio	5 (25.0%)	21 (17.5%)	4 (16.0%)	63 (50.4%)	3 (27.3%)	4 (16.0%)
Medications to treat the infection						
Ibuprofen	3 (15.0%)	11 (9.2%)	4 (16.0%)	19 (15.2%)	1 (9.1%)	3 (12.0%)
Compound paracetamol and amantadine hydrochloride	4 (20.0%)	19 (15.8%)	6 (24.0%)	18 (14.4%)	2 (18.2%)	5 (20.0%)
Chinese patent medicines	10 (50.0%)	70 (58.3%)	14 (56.0%)	95 (76.0%)	5 (45.5%)	17 (68.0%)
Ambroxol	1 (5.0%)	7 (5.8%)	0 (0.0%)	9 (7.2%)	0 (0.0%)	0 (0.0%)
Antibiotics	0 (0.0%)	4 (3.3%)	2 (8.0%)	7 (5.6%)	0 (0.0%)	3 (12.0%)

y, year; %, percentage; d, day; TDM, therapeutic drug monitoring.

### Comparison of different antipsychotic concentrations pre- and post-infection

**Figure 1:**
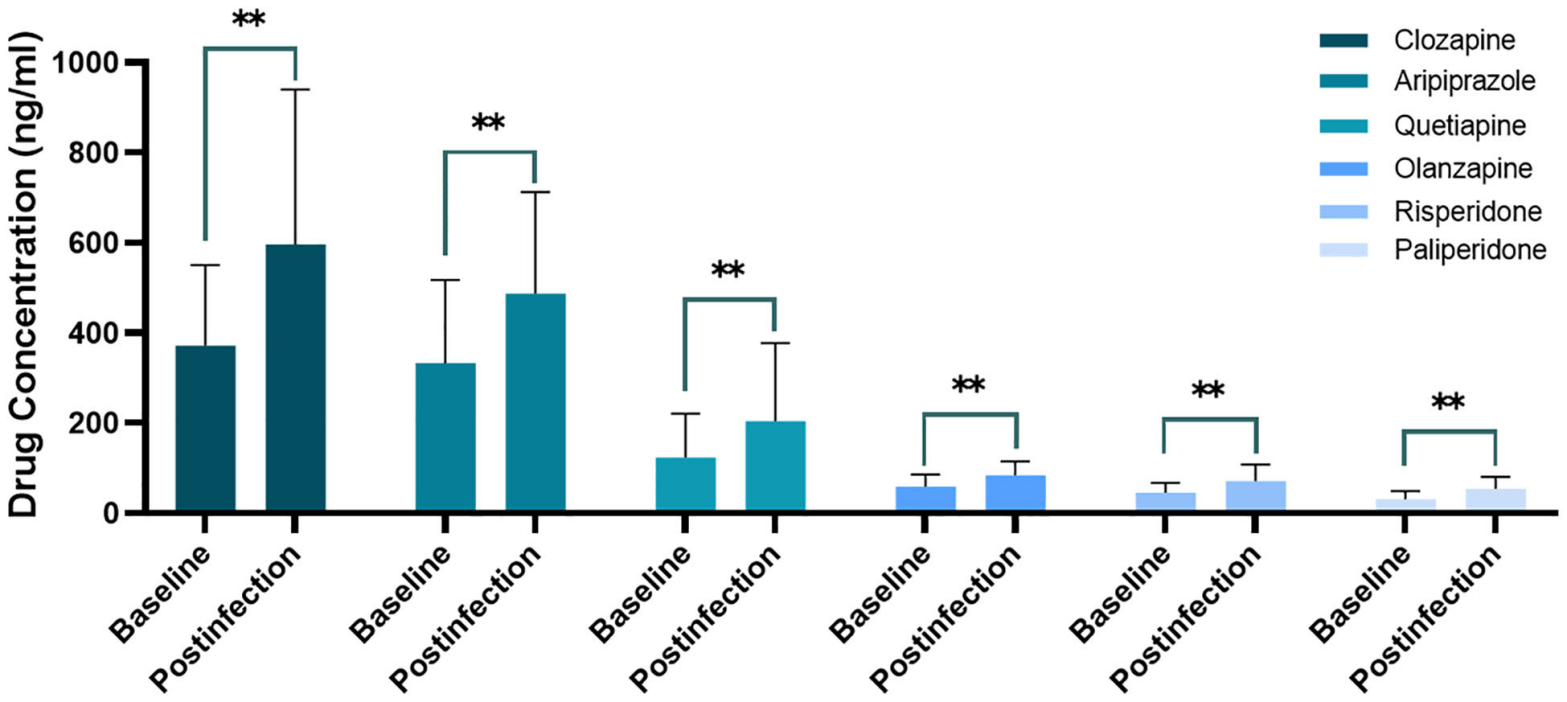
Comparison of different drug pre- and post-coronavirus disease-2019. **P < 0.01.

The concentrations of clozapine (P < 0.001), aripiprazole (P < 0.001), quetiapine (P = 0.005), olanzapine (P < 0.001), risperidone (P < 0.001), and paliperidone (P < 0.001) significantly increased by different degrees post-infection (**Figure 1**).

**Figure 2:**
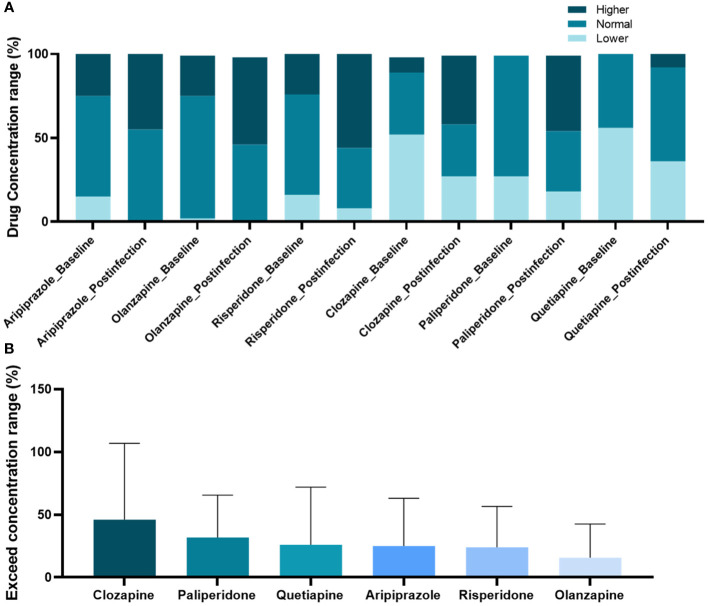
Changes in concentration range of antipsychotic drugs post-coronavirus disease-2019 compared to pre-infection. **(A)** Comparison of antipsychotic drug concentration proportions across three defined levels; **(B)** Ranking of elevated ratios of drug concentration ranges.

According to the AGNP, concentrations of antipsychotic were divided into three levels, subtherapeutic concentrations, within the range of therapeutic concentrations and supratherapeutic concentrations for comparison between pre- and post-infection. The results revealed that the proportion of individuals whose concentrations of clozapine, aripiprazole, quetiapine, olanzapine, risperidone, and paliperidone were supratherapeutic concentrations increased after COVID-19 (**Figure 2A**). However, among patients who used antipsychotic drugs and were infected with COVID-19, the percentage of patients whose clozapine concentration exceeded the pre-infection concentration range was the highest (**Figure 2B**).

### Factors affecting antipsychotic concentrations and dosage adjustments

In the logistic regression model, the results revealed that the drug concentration post-infection significantly correlated with the type of antipsychotic and baseline drug concentrations. Compared with patients receiving quetiapine, those receiving clozapine as the main antipsychotic had an increased risk of elevated clozapine concentrations post-infection (odds ratios [OR] = 12.94, P = 0.0158). Compared with patients within the range of therapeutic concentrations, those with subtherapeutic concentration pre-infection had a reduced risk of elevated concentrations post-infection (OR = 0.08, P < 0.0001), and those with supratherapeutic concentrations pre-infection had an increased risk of elevated concentrations post-infection (OR = 33.45, P < 0.0001). Age, sex, body temperature, smoking status, and concomitant medication were not significantly associated with the changes in drug concentration post-infection. However, the use of Traditional Chinese patent medicines (mainly Lianhua Qingwen Capsules and Jinhua Qinggan Granules; please refer to the **Supplementary Materials** for detailed types of drugs) in patients with mental disorders undergoing symptomatic treatment for COVID-19 was identified as a risk factor for increasing the concentration of antipsychotic drugs (OR = 2.55, P = 0.0042) (**Table 2**).

**Table 2:** Association between drug concentrations post-infection and demographic and clinical factors.

Variables	β	SE	OR	95%CI	*P*
Intercept	–0.296	0.911			0.7455
Age	0.004	0.011	1.00	0.98–1.03	0.7420
Sex					
Female	Ref		1		
Male	0.183	0.180	1.44	0.71–2.92	0.3096
Antipsychotics					
Quetiapine	Ref		1		
Aripiprazole	–0.235	0.581	4.50	0.61–33.07	0.6864
Olanzapine	–0.010	0.314	5.63	0.98–32.37	0.9739
Risperidone	0.339	0.500	7.99	1.08–59.32	0.4978
Clozapine	0.822	0.340	12.94	2.21–75.62	0.0158
Paliperidone	0.823	0.630	12.95	1.43–117.04	0.1916
Baseline_TDM					
Normal	Ref		1		
Lower	–2.819	0.383	0.08	0.04–0.20	< 0.0001
Higher	3.165	0.525	33.45	7.54–148.53	< 0.0001
Temperature	0.079	0.166	1.17	0.61–2.24	0.6315
Smoking	–0.046	0.187	0.91	0.44–1.90	0.8065
Concomitant medications					
Antidepressant	–0.040	0.322	0.92	0.26–3.26	0.9015
Mood stabilizer	0.422	0.248	2.33	0.88–6.14	0.0884
Benzodiazepine	–0.181	0.187	0.70	0.34–1.45	0.3322
Medications to treat the infection					
Ibuprofen	–0.146	0.232	0.75	0.3–1.86	0.5302
Compound paracetamol and amantadine hydrochloride	–0.152	0.202	0.74	0.34–1.63	0.4523
Chinese patent medicines	0.469	0.164	2.55	1.34–4.85	0.0042
Ambroxol	–0.137	0.369	0.76	0.18–3.23	0.7109
Antibiotics	0.665	0.370	3.78	0.89–16.08	0.0721

Ref, reference; TDM, therapeutic drug monitoring.

The results revealed that the pre-infection drug concentration, sex, and antipsychotic type were significantly associated with whether patients underwent major antipsychotic dose adjustment. Among them, men were more likely to require dose adjustments after a drug concentration increase (OR =2.67, P =0.005) (**Table 3**).

**Table 3:** Association between drug dose adjustment post-infection and demographic and clinical factors.

Variables	β	SE	OR	95%CI	*P*
Intercept	–0.746	0.835			0.3717
Age	0.003	0.011	1.00	0.98–1.02	0.7676
Sex					
Female	Ref		1		
Male	0.491	0.175	2.67	1.35–5.29	0.005
Antipsychotics					
Quetiapine	Ref		1		
Aripiprazole	0.060	0.552	1.46	0.27–7.9	0.9131
Olanzapine	–0.776	0.324	0.63	0.15–2.76	0.0166
Risperidone	–1.141	0.577	0.44	0.07–2.85	0.0680
Clozapine	1.567	0.321	6.58	1.57–27.66	< 0.0001
Paliperidone	0.607	0.634	2.52	0.35–17.97	0.3385
Baseline_TDM					
Normal	Ref		1		
Lower	–1.039	0.266	0.31	0.15–0.65	< 0.0001
Higher	0.910	0.279	2.19	0.99–4.81	0.0011
Temperature	0.270	0.167	1.72	0.89–3.31	0.1062
Smoking	0.056	0.189	1.12	0.53–2.35	0.7694
Concomitant medications					
Antidepressant	–0.240	0.290	0.62	0.2–1.93	0.4081
Mood stabilizer	0.242	0.245	1.62	0.62–4.24	0.3232
Benzodiazepine	–0.085	0.177	0.84	0.42–1.69	0.6315
Medications to treat the infection					
Ibuprofen	–0.109	0.217	0.80	0.34–1.88	0.6153
Compound paracetamol and amantadine hydrochloride	–0.151	0.201	0.74	0.34–1.62	0.4506
Chinese patent medicines	0.362	0.161	2.06	1.1–3.88	0.0247
Ambroxol	0.250	0.305	1.65	0.5–5.45	0.4116
Antibiotics	1.009	0.332	7.53	2.05–27.67	0.0024

Ref, reference; TDM, therapeutic drug monitoring.

Patients receiving clozapine were more likely to adjust their dose after a concentration increase (OR = 6.58, P < 0.0001), whereas those using olanzapine were less likely to require dose adjustment after a concentration increase (OR = 0.63, P = 0.0166). Patients with pre-infection subtherapeutic concentration were less likely to require dose adjustment after a drug concentration increase (OR = 0.31, P < 0.0001). Patients with pre-infection supratherapeutic concentrations were more likely to adjust their doses after reaching elevated drug concentrations (OR = 2.19, P = 0.0011). The use of traditional Chinese patent medicines and antibiotics during COVID-19 infection was identified as a risk factor for the reduction or withdrawal of antipsychotic drugs (OR = 2.06, P = 0.0247; OR = 7.53, P = 0.0024, respectively) (**Table 3**).

## Discussion

This study aimed to explore the effects of COVID-19 on antipsychotic concentrations in patients with mental disorders. The concentrations of various antipsychotics increased to varying degrees after COVID-19, which is consistent with the results of previous studies (22). Inflammation can downregulate transporters and drug-metabolizing enzymes, alter plasma protein binding, which in turn affects the absorption, distribution, and clearance of drugs, thereby contributing to an increase in antipsychotic drug serum levels due to a potential slowdown in their metabolism (23–25). Higher concentrations of antipsychotics were associated with increased incidents of somnolence and sedation. Furthermore, there was a higher frequency of reported adverse drug reactions or patient complaints that might be attributed to these heightened levels. Nevertheless, patients’ psychiatric symptoms may seemingly improve “relatively better” due to either the novel coronavirus infection or elevated antipsychotic levels, which could inadvertently lead clinicians to neglect the ongoing management of their psychiatric condition. Consequently, heightened vigilance is imperative for psychiatrists to meticulously monitor antipsychotic concentrations and adverse effects, and dynamically assess changes in mental symptoms in COVID-19-infected psychiatric patients. The inflammatory mediators interleukin-6 (IL-6), tumor necrosis factor-α, and IL-10 are considered the main factors inhibiting drug metabolism enzymes, and they have slightly different effects on different cytochrome p450 (CYP) subtypes (26, 27). The liver is the main target of IL-6, resulting in decreased activity of CYP1A2, CYP2B6, CYP2C8, CYP2C9, CYP2C19, and CYP3A4 (28–31). Among the antipsychotics used in this study, the key enzymes involved in aripiprazole metabolism are CYP2D6 and CYP3A4 (32, 33). The key enzymes in clozapine are CYP1A2, CYP2C19, and CYP3A4 (34, 35). The key enzymes involved in olanzapine are n-glucuronaldehyde transferase, flavin monooxygenase, CYP1A2, and CYP2D6 (18, 36, 37). The key enzymes involved in the action of quetiapine are CYP3A4 and CYP2D6 (28, 37, 38). The key enzymes involved in risperidone and paliperidone are CYP2D6 and CYP3A4 (39). The key enzymes required for the metabolism of various antipsychotics are blocked by IL-6 to varying degrees, and this may be a possible reason for the decline in the metabolism of various antipsychotics after COVID-19. Moreover, the proportion of patients in the clozapine group with a post-infection drug concentration range exceed that of pre-infection was the highest. This may be related to the inhibition of three key metabolic enzymes of clozapine. Additionally, Hefner et al. confirmed that the plasma concentration of clozapine increased significantly more than that of quetiapine and risperidone under inflammatory conditions (23). And in the follow-up, we found that clozapine was the highest reduction or discontinuation rate. Besides more significant increase in drug concentration, it may also be related to more side effects caused by clozapine, including sedation, hypersalivation, and increased appetite, compared to other antipsychotics. Therefore, there is a higher probability that patients may tend to omit some of their daily doses while hospitalized.

We compared the elevated concentrations of the different drugs in pairs, and quetiapine demonstrated a reduced risk of elevated concentrations compared with other antipsychotics; therefore, we used quetiapine as the control in the regression analysis. A significant correlation was identified between the drug concentration post-infection and the type of antipsychotic and drug concentration pre-infection, which is consistent with those of previous studies (26, 40, 41). Our research indicates that the use of traditional Chinese patent medicines among COVID-19 patients with mental disorders may result in elevated concentrations of antipsychotic drugs. However, ibuprofen, compound paracetamol and amantadine hydrochloride, ambroxol, and antibiotics are not associated with an increase in antipsychotic concentrations. It is well known that the use of traditional Chinese patent medicines for treating COVID-19 is widespread in China (42–44). Therefore, this result emphasizes the importance of exercising caution when using them in treating patients with mental disorders who are also infected with COVID-19. Once administered, it is crucial to promptly monitor the concentrations of antipsychotics and adjust the dosage accordingly. Although drug concentration levels are affected by a combination of factors (for example, inconsistencies in treatment regimens for COVID-19 symptoms, concurrent use of other medications, or the presence of physical illnesses may all serve as confounding factors affecting drug concentrations), the most important factor is the antipsychotic itself, including dose, time of use, and type of drug used. When the baseline drug concentration was subtherapeutic concentration, the post-infection drug concentration was less likely to increase, whereas when the baseline drug concentration was supratherapeutic concentrations, the post-infection drug concentration was more likely to increase.

In addition, we did not find obvious correlation between body temperature, smoking and drug concentration. This is inconsistent with previous studies that have demonstrated that drug concentration is also correlated with individual factors such as age, sex, and smoking (45–48). Studies have reported that polycyclic aromatic hydrocarbons in tobacco smoke can affect CYP1A2 (49, 50) and smokers who take the same dose of clozapine orally have lower concentrations than non-smokers (51–53). All patients in this study were hospitalized. To reduce the incidence of COVID-19 during the epidemic period in our hospital, cigarettes were totally prohibited from being brought into the ward, so patients couldn’t obtain cigarettes during their stay. Therefore, the smokers in this study (94 patients) were in a “quitting smoking” stage when the drug concentration was measured at baseline and post-infection. In a study of 12 heavy smokers by Faber et al., CYP1A2 clearance decreased by 12% on day 1 after quitting and reached a steady state on day 6, with an average clearance 36% lower than before quitting (54). The smokers in this study quit smoking for at least 1 week before the baseline drug concentration was measured, and the influence of smoking and quitting on the concentration was significantly reduced. This may explain why the drug concentration in this study did not correlate with smoking. The increase in drug concentration caused by fever was mainly related to the loss of body fluid. Altogether, 214 patients with fever were included in this study, including 36 cases with high fever (body temperature ≥ 38.5°C) and 178 cases with low fever (37.3°C ≤ body temperature < 38.5°C). Timely fluid rehydration was administered during clinical treatment. Therefore, the reasons for the lack of correlation between body temperature and drug concentration may be as follows. First, the low fever state rarely causes body fluid loss which can lead to increased drug concentration, and the sample size of patients with high fever was small. The sample size could be expanded to further monitor the influence of high and low fever on drug concentration. Second, timely and effective rehydration treatment may compensate for the loss of body fluids caused by fever, resulting in little change in drug concentration.

The regression analysis revealed that pre-infection drug concentration levels, sex, and antipsychotic type were significantly associated with whether patients underwent major antipsychotic dose adjustments. Among them, patients using olanzapine as the primary antipsychotic were less likely to adjust their dose after a drug concentration increase (OR = 0.63, P = 0.0166). Patients receiving clozapine as their primary antipsychotic were more likely to adjust their doses after an increase in drug concentration (OR = 6.58, P < 0.0001). The results for other drugs were not statistically significant. Although the olanzapine concentration also increased post-infection, IL-6 may mainly affected the P450 enzyme system. In addition to CYP1A2 and CYP2D6, olanzapine can complete its metabolic processes via n-glucuronaldehyde transferase, which is weakly affected by inflammatory mediators (55). This also explains why the concentration of olanzapine varied less in patients receiving olanzapine after COVID-19.

### Strengths and limitations

The key strengths of our study include the coverage of various antipsychotics. For the first time, we explored the relationship between the concentration of most frequently-used antipsychotics, including clozapine, and COVID-19. The findings of this study can help guide the use of antipsychotics in patients in whom mental disorders are comorbid with COVID-19. This study also has significance as a reference for changes in drug concentrations caused by other viral or bacterial infections.

A major limitation of this study is that we did not exclude changes in drug concentrations caused by drug interactions in the case of multiple antipsychotics, which may explain the differences between this study and other studies. Another limitation is that COVID-19, as a newly discovered virus, causes infections involving the respiratory, digestive, cardiovascular, nervous, and other systems (56, 57). The influence of the patients’ physical conditions on the concentration of antipsychotic drugs has not been thoroughly examined.

## Conclusion

The concentration of antipsychotics (clozapine, aripiprazole, quetiapine, olanzapine, risperidone, and paliperidone) increased in patients with mental disorders after COVID-19, with clozapine showing the most significant increase. When adjusting the doses of antipsychotics for mental disorders following COVID-19 infection, factors such as the patient’s sex, type of antipsychotic, pre-infection drug concentration, and type of drug used for COVID-19 should be considered. Psychiatrists should pay special attention to the use of combined prescriptions when dealing with patients with mental disorders infected with COVID-19, especially when combining traditional Chinese patent medicines, and should closely monitor the concentration of antipsychotic drugs.

## Data availability statement

The raw data supporting the conclusions of this article will be made available by the authors, without undue reservation.

## Ethics statement

The studies involving humans were approved by Ethics Committee of Beijing HuiLongGuan Hospital. The studies were conducted in accordance with the local legislation and institutional requirements. The participants provided their written informed consent to participate in this study. Written informed consent was obtained from the individual(s) for the publication of any potentially identifiable images or data included in this article.

## Author contributions

RY: Investigation, Validation, Writing – original draft. J-LW: Investigation, Validation, Writing – original draft. C-QP: Investigation, Validation, Writing – original draft. T-HW: Investigation, Validation, Writing – original draft. X-QZ: Data curation, Formal analysis, Methodology, Writing – review & editing. S-JZ: Conceptualization, Project administration, Supervision, Writing – review & editing.

## References

[1] Toubasi AA AbuAnzeh RB Tawileh HBA Aldebei RH Alryalat SAS. A meta-analysis: the mortality and severity of covid-19 among patients with mental disorders. Psychiatry Res. (2021) 299:113856. doi: 10.1016/j.psychres.2021.113856 33740483 PMC7927594

[2] Fornaro M De Prisco M Billeci M Ermini E Young AH Lafer B. Implications of the covid-19 pandemic for people with bipolar disorders: A scoping review. J Affect Disord. (2021) 295:740–51. doi: 10.1016/j.jad.2021.08.091 PMC8416293 34517248

[3] Vai B Mazza MG. Antipsychotics and covid-19 outcomes-the potential role of the clinical setting? JAMA Netw Open. (2022) 5:e2210749. doi: 10.1001/jamanetworkopen.2022.10749 35522287

[4] Sparasci O Bhui K. Impact of covid-19 on mental health research: is this the breaking point? Br J Psychiatry. (2022) 220(5):1–3. doi: 10.1192/bjp.2022.8 PMC7612706 35172915

[5] Pinkham AE Ackerman RA Depp CA Harvey PD Moore RC. A longitudinal investigation of the effects of the covid-19 pandemic on the mental health of individuals with pre-existing severe mental illnesses. Psychiatry Res. (2020) 294:113493. doi: 10.1016/j.psychres.2020.113493 33038789 PMC7528831

[6] Morillo-González J Hernández-Huerta D Guillama-Henríquez A Correa-Palacio A Pereira-Nogueira P. Beyond the respiratory system: A case report highlighting the impact of covid-19 in mental illness and its physical consequences. J Clin Psychiatry. (2020) 81:20l13465. doi: 10.4088/JCP.20l13465 32558406

[7] Urban AE Cubała WJ. Therapeutic drug monitoring of atypical antipsychotics. Psychiatr Pol. (2017) 51:1059–77. doi: 10.12740/PP/65307 29432503

[8] Cranshaw T Harikumar T. Covid-19 infection may cause clozapine intoxication: case report and discussion. Schizophr Bull. (2020) 46:751. doi: 10.1093/schbul/sbaa070 32435811 PMC7313764

[9] Arrojo-Romero M Codesido-Barcala MR de Leon J. A covid-19 outbreak in a spanish long-term psychiatric hospital led to infections in 6 clozapine patients: elevations in their plasma clozapine levels. Rev Psiquiatr Salud Ment (Engl Ed). (2022) 15:290–2. doi: 10.1016/j.rpsmen.2022.06.010 PMC9233870 35782582

[10] Veerman SRT Bogers J Cohen D Schulte PFJ. Covid-19: risks, complications, and monitoring in patients on clozapine. Pharmacopsychiatry. (2022) 55:48–56. doi: 10.1055/a-1562-2521 34470068

[11] Nemani K Williams SZ Olfson M Leckman-Westin E Finnerty M Kammer J. Association between the use of psychotropic medications and the risk of covid-19 infection among long-term inpatients with serious mental illness in a new york state-wide psychiatric hospital system. JAMA Netw Open. (2022) 5:e2210743. doi: 10.1001/jamanetworkopen.2022.10743 35522282 PMC9077485

[12] Okusaga OO Mitchell BG Bernard JD Walder A. Clozapine is associated with higher covid-19 infection rate in veterans with schizophrenia or schizoaffective disorder. J Clin Psychiatry. (2021) 82:21br14028. doi: 10.4088/JCP.21br14028 34428355

[13] Siskind D Honer WG Clark S Correll CU Hasan A Howes O. Consensus statement on the use of clozapine during the covid-19 pandemic. J Psychiatry Neurosci. (2020) 45:222–3. doi: 10.1503/jpn.200061 PMC7828973 32297722

[14] Campitelli MA Bronskill SE Maclagan LC Harris DA Cotton CA Tadrous M. Comparison of Medication Prescribing before and after the Covid-19 Pandemic among Nursing Home Residents in Ontario, Canada. JAMA Netw Open. (2021) 4:e2118441. doi: 10.1001/jamanetworkopen.2021.18441 34338794 PMC8329744

[15] de Leon J Ruan CJ Schoretsanitis G De Las Cuevas C. A rational use of clozapine based on adverse drug reactions, pharmacokinetics, and clinical pharmacopsychology. Psychother Psychosom. (2020) 89:200–14. doi: 10.1159/000507638 PMC7206357 32289791

[16] Tio N Schulte PFJ Martens HJM. Clozapine intoxication in covid-19. Am J Psychiatry. (2021) 178:123–7. doi: 10.1176/appi.ajp.2020.20071039 33517757

[17] Mauri MC Paletta S Di Pace C Reggiori A Cirnigliaro G Valli I. Clinical pharmacokinetics of atypical antipsychotics: an update. Clin Pharmacokinet. (2018) 57:1493–528. doi: 10.1007/s40262-018-0664-3 29915922

[18] Soria-Chacartegui P Villapalos-García G Zubiaur P Abad-Santos F Koller D. Genetic polymorphisms associated with the pharmacokinetics, pharmacodynamics and adverse effects of olanzapine, aripiprazole and risperidone. Front Pharmacol. (2021) 12:711940. doi: 10.3389/fphar.2021.711940 34335273 PMC8316766

[19] China TPsRo. Diagnosis and Treatment Protocol for Novel Coronavirus Pneumonia (Trial Version 9) (2022). Available online at: https://www.gov.cn/zhengce/zhengceku/2022-03/15/5679257/files/49854a49c7004f4ea9e622f3f2c568d8.pdf.

[20] Organization WH. Coronavirus Disease (Covid-19) (2023). Available online at: https://www.who.int/news-room/fact-sheets/detail/coronavirus-disease-(covid-19).

[21] Hiemke C Bergemann N Clement HW Conca A Deckert J Domschke K. Consensus guidelines for therapeutic drug monitoring in neuropsychopharmacology: update 2017. Pharmacopsychiatry. (2018) 51:9–62. doi: 10.1055/s-0043-116492 28910830

[22] Pan S Li W Shi L Li Y Wang X Zhou Y. Relationship between C-reactive protein and antipsychotics levels in schizophrenic patients infected with covid-19. J Psychiatr Res. (2024) 170:297–301. doi: 10.1016/j.jpsychires.2024.01.002 38185075

[23] Hefner G Shams ME Unterecker S Falter T Hiemke C. Inflammation and psychotropic drugs: the relationship between C-reactive protein and antipsychotic drug levels. Psychopharmacol (Berl). (2016) 233:1695–705. doi: 10.1007/s00213-015-3976-0 26032842

[24] Scherf-Clavel M Weidner A Deckert J Menke A Unterecker S. Pathological concentration of C-reactive protein is correlated to increased concentrations of quetiapine, but not of risperidone, olanzapine and aripiprazole in a naturalistic setting. Pharmacopsychiatry. (2020) 53:30–5. doi: 10.1055/a-0869-8053 30913567

[25] Moschny N Hefner G Grohmann R Eckermann G Maier HB Seifert J. Therapeutic drug monitoring of second- and third-generation antipsychotic drugs-influence of smoking behavior and inflammation on pharmacokinetics. Pharmaceuticals (Basel). (2021) 14(6):514. doi: 10.3390/ph14060514 34071813 PMC8230242

[26] Shah RR Smith RL. Inflammation-induced phenoconversion of polymorphic drug metabolizing enzymes: hypothesis with implications for personalized medicine. Drug Metab Dispos. (2015) 43:400–10. doi: 10.1124/dmd.114.061093 25519488

[27] Dickmann LJ Patel SK Wienkers LC Slatter JG. Effects of interleukin 1β (Il-1β) and il-1β/interleukin 6 (Il-6) combinations on drug metabolizing enzymes in human hepatocyte culture. Curr Drug Metab. (2012) 13:930–7. doi: 10.2174/138920012802138642 22475267

[28] Klein C Wüstefeld T Assmus U Roskams T Rose-John S Müller M. The il-6-gp130-stat3 pathway in hepatocytes triggers liver protection in T cell-mediated liver injury. J Clin Invest. (2005) 115:860–9. doi: 10.1172/JCI23640 PMC1059450 15761498

[29] Klein M Thomas M Hofmann U Seehofer D Damm G Zanger UM. A systematic comparison of the impact of inflammatory signaling on absorption, distribution, metabolism, and excretion gene expression and activity in primary human hepatocytes and heparg cells. Drug Metab Dispos. (2015) 43:273–83. doi: 10.1124/dmd.114.060962 25480923

[30] Dickmann LJ Patel SK Rock DA Wienkers LC Slatter JG. Effects of interleukin-6 (Il-6) and an anti-il-6 monoclonal antibody on drug-metabolizing enzymes in human hepatocyte culture. Drug Metab Dispos. (2011) 39:1415–22. doi: 10.1124/dmd.111.038679 21555507

[31] Rubin K Janefeldt A Andersson L Berke Z Grime K Andersson TB. Heparg cells as human-relevant *in vitro* model to study the effects of inflammatory stimuli on cytochrome P450 isoenzymes. Drug Metab Dispos. (2015) 43:119–25. doi: 10.1124/dmd.114.059246 25371393

[32] Waade RB Christensen H Rudberg I Refsum H Hermann M. Influence of comedication on serum concentrations of aripiprazole and dehydroaripiprazole. Ther Drug Monit. (2009) 31:233–8. doi: 10.1097/FTD.0b013e3181956726 19142178

[33] Hendset M Hermann M Lunde H Refsum H Molden E. Impact of the cyp2d6 genotype on steady-state serum concentrations of aripiprazole and dehydroaripiprazole. Eur J Clin Pharmacol. (2007) 63:1147–51. doi: 10.1007/s00228-007-0373-6 17828532

[34] Jaquenoud Sirot E Knezevic B Morena GP Harenberg S Oneda B Crettol S. Abcb1 and cytochrome P450 polymorphisms: clinical pharmacogenetics of clozapine. J Clin Psychopharmacol. (2009) 29:319–26. doi: 10.1097/JCP.0b013e3181acc372 19593168

[35] Olesen OV Linnet K. Contributions of five human cytochrome P450 isoforms to the N-demethylation of clozapine *in vitro* at low and high concentrations. J Clin Pharmacol. (2001) 41:823–32. doi: 10.1177/00912700122010717 11504269

[36] Mao JH Han L. Significant predictors for olanzapine pharmacokinetics: A systematic review of population pharmacokinetic studies. Expert Rev Clin Pharmacol. (2023) 16(6):575–88. doi: 10.1080/17512433.2023.2219055 37231707

[37] Le Daré B Ferron PJ Allard PM Clément B Morel I Gicquel T. New insights into quetiapine metabolism using molecular networking. Sci Rep. (2020) 10:19921. doi: 10.1038/s41598-020-77106-x 33199804 PMC7669884

[38] Bakken GV Rudberg I Christensen H Molden E Refsum H Hermann M. Metabolism of quetiapine by cyp3a4 and cyp3a5 in presence or absence of cytochrome B5. Drug Metab Dispos. (2009) 37:254–8. doi: 10.1124/dmd.108.023291 19022943

[39] Xiang Q Zhao X Zhou Y Duan JL Cui YM. Effect of cyp2d6, cyp3a5, and mdr1 genetic polymorphisms on the pharmacokinetics of risperidone and its active moiety. J Clin Pharmacol. (2010) 50:659–66. doi: 10.1177/0091270009347867 20332423

[40] Stingl JC Brockmöller J Viviani R. Genetic variability of drug-metabolizing enzymes: the dual impact on psychiatric therapy and regulation of brain function. Mol Psychiatry. (2013) 18:273–87. doi: 10.1038/mp.2012.42 22565785

[41] Brockmöller J Kirchheiner J Meisel C Roots I. Pharmacogenetic diagnostics of cytochrome P450 polymorphisms in clinical drug development and in drug treatment. Pharmacogenomics. (2000) 1:125–51. doi: 10.1517/14622416.1.2.125 11256586

[42] Hu K Guan WJ Bi Y Zhang W Li L Zhang B. Efficacy and safety of lianhuaqingwen capsules, a repurposed chinese herb, in patients with coronavirus disease 2019: A multicenter, prospective, randomized controlled trial. Phytomedicine. (2021) 85:153242. doi: 10.1016/j.phymed.2020.153242 33867046 PMC7229744

[43] Liang C Hui N Liu Y Qiao G Li J Tian L. Insights into forsythia honeysuckle (Lianhuaqingwen) capsules: A chinese herbal medicine repurposed for covid-19 pandemic. Phytomed Plus. (2021) 1:100027. doi: 10.1016/j.phyplu.2021.100027 35399819 PMC7833308

[44] Xu Q Song K Clifford SP Kong M Huang J. Meta-analysis of traditional chinese medicine lianhua qingwen in the treatment of coronavirus disease 2019. J Anesth Transl Med. (2023) 2:20–6. doi: 10.58888/2957-3912-2023-06-21 PMC10878371 38380434

[45] Zang YN Dong F Li AN Wang CY. Correction to: the impact of smoking, sex, infection, and comedication administration on oral olanzapine: A population pharmacokinetic model in chinese psychiatric patients. Eur J Drug Metab Pharmacokinet. (2021) 46(3):373–4. doi: 10.1007/s13318-021-00680-6 33743172

[46] Kawada T. Factors affecting serum olanzapine concentration. Ther Drug Monit. (2021) 43:301. doi: 10.1097/FTD.0000000000000852 33298745

[47] Gex-Fabry M Balant-Gorgia AE Balant LP. Therapeutic drug monitoring of olanzapine: the combined effect of age, gender, smoking, and comedication. Ther Drug Monit. (2003) 25:46–53. doi: 10.1097/00007691-200302000-00007 12548144

[48] Zhang Y Wilkins JM Bessette LG York C Wong V Lin KJ. Antipsychotic medication use among older adults following infection-related hospitalization. JAMA Netw Open. (2023) 6:e230063. doi: 10.1001/jamanetworkopen.2023.0063 36800180 PMC9938426

[49] Meyer JM. Individual changes in clozapine levels after smoking cessation: results and a predictive model. J Clin Psychopharmacol. (2001) 21:569–74. doi: 10.1097/00004714-200112000-00005 11763003

[50] Tsuda Y Saruwatari J Yasui-Furukori N. Meta-analysis: the effects of smoking on the disposition of two commonly used antipsychotic agents, olanzapine and clozapine. BMJ Open. (2014) 4:e004216. doi: 10.1136/bmjopen-2013-004216 PMC3948577 24595134

[51] Madsen HKL Gulløv M Farver-Vestergaard I Hjort P Nielsen LP Løkke A. Smoking cessation and drug interactions. Ugeskr Laeger. (2022) 184:V02220117. 36065858

[52] Scherf-Clavel M Samanski L Hommers LG Deckert J Menke A Unterecker S. Analysis of smoking behavior on the pharmacokinetics of antidepressants and antipsychotics: evidence for the role of alternative pathways apart from cyp1a2. Int Clin Psychopharmacol. (2019) 34:93–100. doi: 10.1097/YIC.0000000000000250 30557209

[53] Wagner E McMahon L Falkai P Hasan A Siskind D. Impact of smoking behavior on clozapine blood levels - a systematic review and meta-analysis. Acta Psychiatr Scand. (2020) 142(6):456–66. doi: 10.1111/acps.13228 32869278

[54] Faber MS Fuhr U. Time response of cytochrome P450 1a2 activity on cessation of heavy smoking. Clin Pharmacol Ther. (2004) 76:178–84. doi: 10.1016/j.clpt.2004.04.003 15289794

[55] Lévesque E Beaulieu M Guillemette C Hum DW Bélanger A. Effect of interleukins on ugt2b15 and ugt2b17 steroid uridine diphosphate-glucuronosyltransferase expression and activity in the lncap cell line. Endocrinology. (1998) 139:2375–81. doi: 10.1210/endo.139.5.6001 9564848

[56] Umakanthan S Sahu P Ranade AV Bukelo MM Rao JS Abrahao-MaChado LF. Origin, transmission, diagnosis and management of coronavirus disease 2019 (Covid-19). Postgrad Med J. (2020) 96:753–8. doi: 10.1136/postgradmedj-2020-138234 PMC10016932 32563999

[57] Safiabadi Tali SH LeBlanc JJ Sadiq Z Oyewunmi OD Camargo C Nikpour B. Tools and techniques for severe acute respiratory syndrome coronavirus 2 (Sars-cov-2)/covid-19 detection. Clin Microbiol Rev. (2021) 34(3):e00228-20. doi: 10.1128/CMR.00228-20 33980687 PMC8142517

