# Supplementary material for: Increased antipsychotic drug concentration in hospitalized patients with mental disorders following COVID-19 infection: a call for attention
Source: Front Psychiatry. 2024 Jul 15;15:1421370. doi: 10.3389/fpsyt.2024.1421370 (PMC11284031; doi:10.3389/fpsyt.2024.1421370)
Supplement: Supplementary file 1 [file Table_1.docx]

**Table S1** Types of traditional Chinese medicines and their metabolic pathways.

| **Chinese patent medicines** | **Metabolism pathway** |
| --- | --- |
| Lianhua Qingwen Capsules | Hepatically cleared |
| Jinhua Qinggan Granules | Hepatically cleared |
| Compound Licorice Oral Solution | Hepatically cleared |
| Qingkailing Oral Liquid | Hepatically cleared |
| Cold and Fever Granules | Hepatically cleared |
| Pulmonary cough mixture | Hepatically cleared |
| Tangerine Red Pill for Cough Relief | Hepatically cleared |
